# Analysis of viral integration reveals new insights of oncogenic mechanism in HBV-infected intrahepatic cholangiocarcinoma and combined hepatocellular-cholangiocarcinoma

**DOI:** 10.1007/s12072-022-10419-3

**Published:** 2022-09-20

**Authors:** Linghao Zhao, Yuyouye Wang, Tao Tian, Xinjie Rao, Wei Dong, Jinmin Zhang, Yuan Yang, Qifei Tao, Fang Peng, Chenhang Shen, Songbo Wang, Hui Liu, Xi Zeng, Weiping Zhou

**Affiliations:** 1 Shanghai Eastern Hepatobiliary Surgery Hospital, Naval Medical University, Shanghai, 200438 China; 2 Agricultural Bioinformatics Key Laboratory of Hubei Province and Hubei Engineering Technology Research Center of Agricultural Big Data, 3D Genomics Research Center, College of Informatics, Huazhong Agricultural University, Wuhan, 430070 China; 3 Bio-Intelligence Co. Ltd, Shenzhen, 518000 China

**Keywords:** Combined hepatocellular-cholangiocarcinoma, Intrahepatic cholangiocarcinoma, Hepatitis B virus, HBV integration

## Abstract

**Background:**

Integration of HBV DNA into the human genome could progressively contribute to hepatocarcinogenesis. Both intrahepatic cholangiocarcinoma (ICC) and combined hepatocellular-cholangiocarcinoma (CHC) are known to be associated with HBV infection. However, the integration of HBV and mechanism of HBV-induced carcinogenesis in ICC and CHC remains unclear.

**Methods:**

41 patients with ICC and 20 patients with CHC were recruited in the study. We conducted HIVID analysis on these 61 samples to identify HBV integration sites in both the tumor tissues and adjacent non-tumor liver tissues. To further explore the effect of HBV integration on gene alteration, we selected paired tumors and adjacent non-tumor liver tissues from 3 ICC and 4 CHC patients for RNA-seq and WGS.

**Results:**

We detected 493 HBV integration sites in ICC patients, of which 417 were from tumor samples and 76 were from non-tumor samples. And 246 HBV integration sites were detected in CHC patients, of which 156 were located in the genome of tumor samples and 90 were in non-tumor samples. Recurrent HBV integration events were detected in ICC including *TERT*, *ZMAT4*, *MET*, *ANKFN1*, *PLXNB2*, and in CHC like *TERT*, *ALKBH5*. Together with our established data of HBV-infected hepatocellular carcinoma, we found that HBV preferentially integrates into the specific regions which may affect the gene expression and regulation in cells and involved in carcinogenesis. We further performed genomic and transcriptomic sequencing of three ICC and four CHC patients, and found that HBV fragments could integrate near some important oncogene like *TERT*, causing large-scale genome variations on nearby genomic sequences, and at the same time changing the expression level of the oncogenes.

**Conclusion:**

Comparative analysis demonstrates numerous newly discovered mutational events in ICC and CHC resulting from HBV insertions in the host genome. Our study provides an in-depth biological and clinical insights into HBV-induced ICC and CHC.

**Supplementary Information:**

The online version contains supplementary material available at 10.1007/s12072-022-10419-3.

## Background

Infection with the human hepatitis B virus (HBV) is one of the most widespread causes of liver cirrhosis and primary liver cancer (PLC) [1, 2]. Many studies highlight that the integration of HBV DNA into the human genome could progressively contribute to hepatocarcinogenesis [3]. HBV DNA integration appears to occur in early stage of infection and causes genetic damage and chromosomal instability, which is known to be selectively advantageous for tumor progression [4, 5].

Intrahepatic cholangiocarcinoma (ICC) is the second most frequent PLC with poor prognosis after hepatocellular carcinoma (HCC), which accounts for 5–30% of all primary liver malignancies [6–8]. The cell origin of cholangiocarcinoma is still a matter of debate. Over the past decades, the incidence and mortality rates of ICC have increased globally, indicating that ICC has become a growing clinical problem. Accumulating evidence indicates that chronic HBV infection is noteworthy associated with an increased risk of ICC development and suggests an etiological role of HBV even in the development of this tumor [9–14]. It is worth mentioning that the International Agency for Research on Cancer has recently identified ICC as an additional tumor positively linked to HBV.

Combined hepatocellular-cholangiocarcinoma (CHC) is a rare malignant primary hepatic tumor defined as one that contains unequivocal intimately mixed elements of both hepatocellular carcinoma (HCC) and cholangiocarcinoma (CC), which represents 0.4–14.2% of primary liver malignancies [15]. Patients with CHC, especially in Asian countries, are frequently accompanied by liver cirrhosis and chronic HBV infection [16]. Although the World Health Organization (WHO) recognizes CHC as a distinct subtype of hepatic malignancy, the diagnosis, prognosis, and treatment of this neoplasm remain ill-defined. It is also reported that chronic infection with HBV is a risk factor of CHC development [17, 18].

Several previous studies have shown that ICC and CHC may originate from hepatocytes [19–22]. In addition, the finding of viral DNA integration into hepatocytes is known to be involved in cancerogenesis [23]. Thus, as is the case for HCC, HBV infection or integration may synergically elevate the risk of both liver cancers. However, the mechanism of HBV-induced ICC and CHC carcinogenesis remains unclear and poorly characterized, although the role of HBV integrations in HCC development has been well characterized [24–27] In the current study, we applied a High-throughput Viral Integration Detection (HIVID) protocol[28] to identify the HBV integration sites in both ICC and CHC genome, and to characterize the molecular status in tumor and non-tumor liver specimens from these patients.

## Methods

### Sample collection

All samples were obtained from Eastern Hepatobiliary Surgery Hospital (EHBH), Shanghai, between 2009 and 2013. Tumor tissues and paired adjacent non-tumor tissue were resected from patients undergoing primary hepatectomy by experienced surgeons. Specimens were immediately cryopreserved in − 80 °C following resection. All operations were carried out carefully to avoid contamination between samples. Furthermore, pathologists performed histological diagnosis of ICC and CHC. The Institutional Review Board of EHBH has approved the collection and use of these samples.

### Extraction of DNA and RNA for capture sequencing, whole-genome sequencing (WGS), and RNA sequencing (RNA-seq)

Genomic DNA Mini Kit (Invitrogen, Life Technologies) and Total RNA Isolation Kit (Ambion, Life Technologies) were used to extract DNA and RNA from paired tumor and adjacent non-tumor samples. Nanodrop 2000 was used to measure the concentration and quality of RNA in samples. Then, the extracted DNA and RNA were processed to HBV capture sequencing, whole-genome sequencing (WGS), and RNA sequencing (RNA-seq).

### HBV capture experiment and sequencing

To capture the sequence of HBV in samples, the probes were designed and produced by MyGenostics according to the genomes of eight types of HBV subtypes (A, B, C, D, E, F, G, and H). Genomic DNA in human cells was sheared to be fragments of around 300 bp by Covaris E-210 (Covaris, Inc., Woburn, MA). These fragments were purified, end blunted, ‘A’ tailed, adaptor ligated. Next, the DNA fragments and HBV probes were hybridized according to the instruction of Target Enrichment Protocol (GenCapTM Enrichment, MyGenostics, China) at the condition of 65 °C for 24 h. These eluted fragments were amplified by PCR to construct the sequencing library, the concentration of which was assessed by Bioanalyzer 2100. The library was further sequenced by HiSeq 2000 sequencer (Illumina Inc., San Diego, CA) with mode of paired end and read length of 100 bp.

### Identification of HBV integration

HIVID was used to detect the sites of HBV integrations into the human genome and the sites of breakpoints in HBV genome [29]. Briefly speaking, the DNA fragments that could map both on human and HBV genome were conducted to predict the locations of virus integration events. The reads alignment results with mapping quality less than 1 were filtered out. The micro-homology between HBV and human genome was tolerant in the calling of integration sites. The integration events covered by only one DNA fragment were discarded. The supporting reads’ number was normalized by sequencing depth and the integration sites with normalized value no less than 2 were retained for subsequent analysis.

### Analysis of RNA-seq and WGS data

The gene expression levels were obtained from RNA-seq data by hisat2-stringtie pipeline [30]. The WGS data were mapped to reference genome of human and HBV by Burrows–Wheeler Alignment (BWA) tool [31]. The coverage depth each nucleotide on reference genome was determined by Samtools based on the alignment results.

## Results

### The characteristics of integration in the human genome

**Fig. 1:**
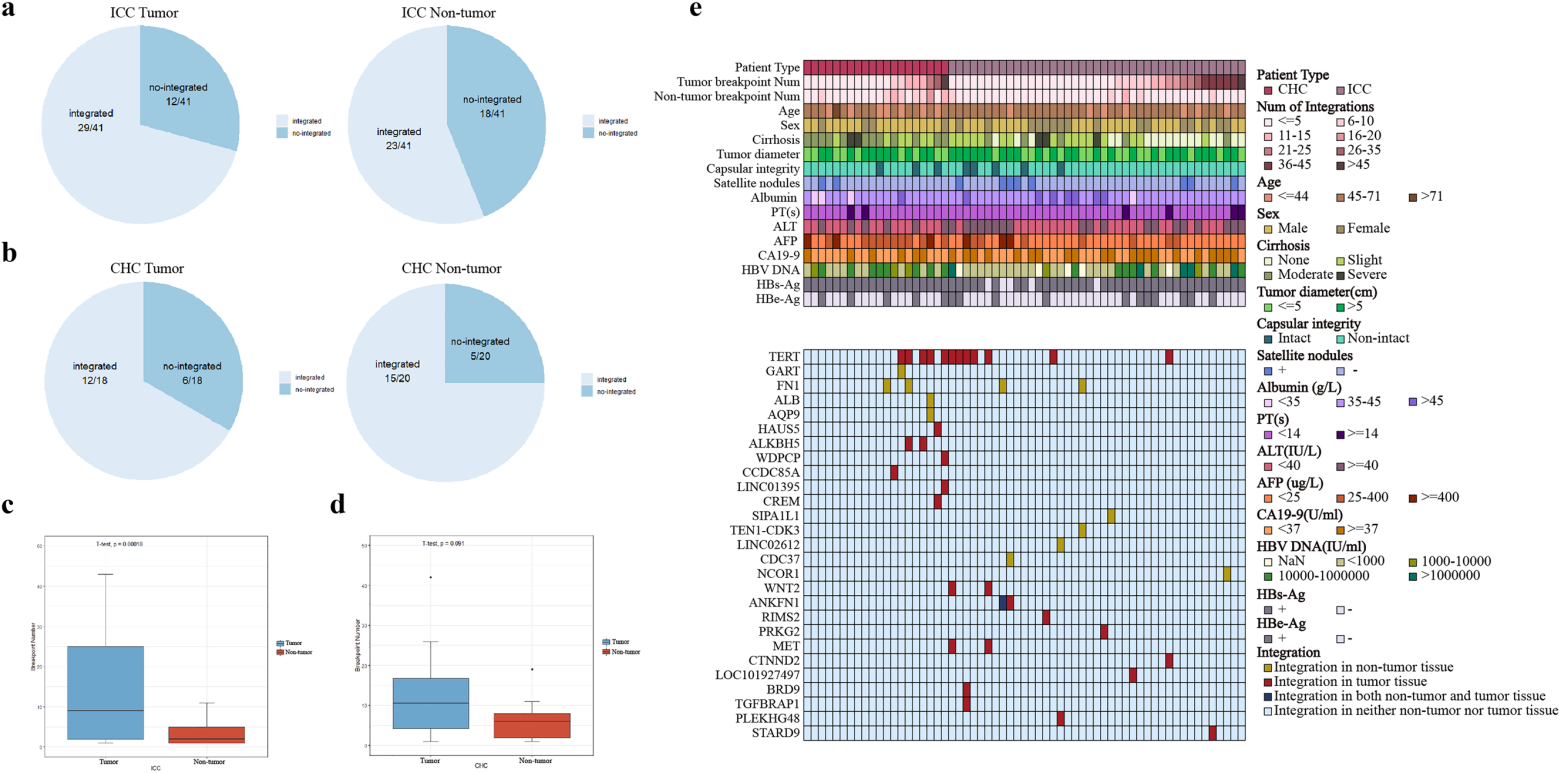
HBV integrations’ distribution in our samples. **a** HBV integration rate in tumor and non-tumor tissues of ICC samples; **b** HBV integration rate in tumor and non-tumor tissues of CHC samples; **c** the comparison of HBV breakpoint numbers between tumor and non-tumor tissues in ICC; **d** the comparison of HBV breakpoint numbers between tumor and non-tumor tissues in CHC; **e** the overview of clinical indicators, number of HBV integrations in each sample, and HBV integrations profiles in the genes of recurrent genes of high frequency

In this study, we collected the tumor tissues and adjacent non-tumor liver tissues of 41 patients with ICC and 20 patients with CHC, and compiled the clinical and pathological data information of these samples (Table S1, Fig. 1e). To identify HBV integration sites in the liver cancer genome and study the virus–host interaction, we conducted HIVID analysis on these 61 samples. With HBV capture sequencing, HBV integration sites in the host genome were effectively detected at 1 base-pair resolution. We detected 493 HBV integration sites in ICC patients, of which 417 were from tumor samples and 76 were from non-tumor samples. And 246 HBV integration sites were detected in CHC patients, of which 156 were located in the genome of tumor samples and 90 were in non-tumor samples (Table S2–S5). Next, we analyzed the characteristics of HBV integration in tumor and non-tumor tissues. The results show that the occurrence ratio of HBV integration is higher in tumor (29/41) than non-tumor tissue (23/41) for ICC patients (70.7% vs 56.1%). While for CHC patients, the HBV integration ratio is a bit lower in tumor (12/18, 66.7%) than non-tumor tissue (15/20, 75%) (Fig. 1a, b). Furthermore, in ICC patients, the average number of integration sites in tumor is significantly higher than in non-tumor tissue (13.45 vs 3.3, *p* value = 0.00018, Student’s *T* test). Similar results were found in CHC, where the average number of integration sites in tumor is higher than that in non-tumor tissue (13.7 vs 6.4, *p* value = 0.091, Student’s *T* test), although the difference is not statistically significant (Fig. 1c, d).

**Fig. 2:**
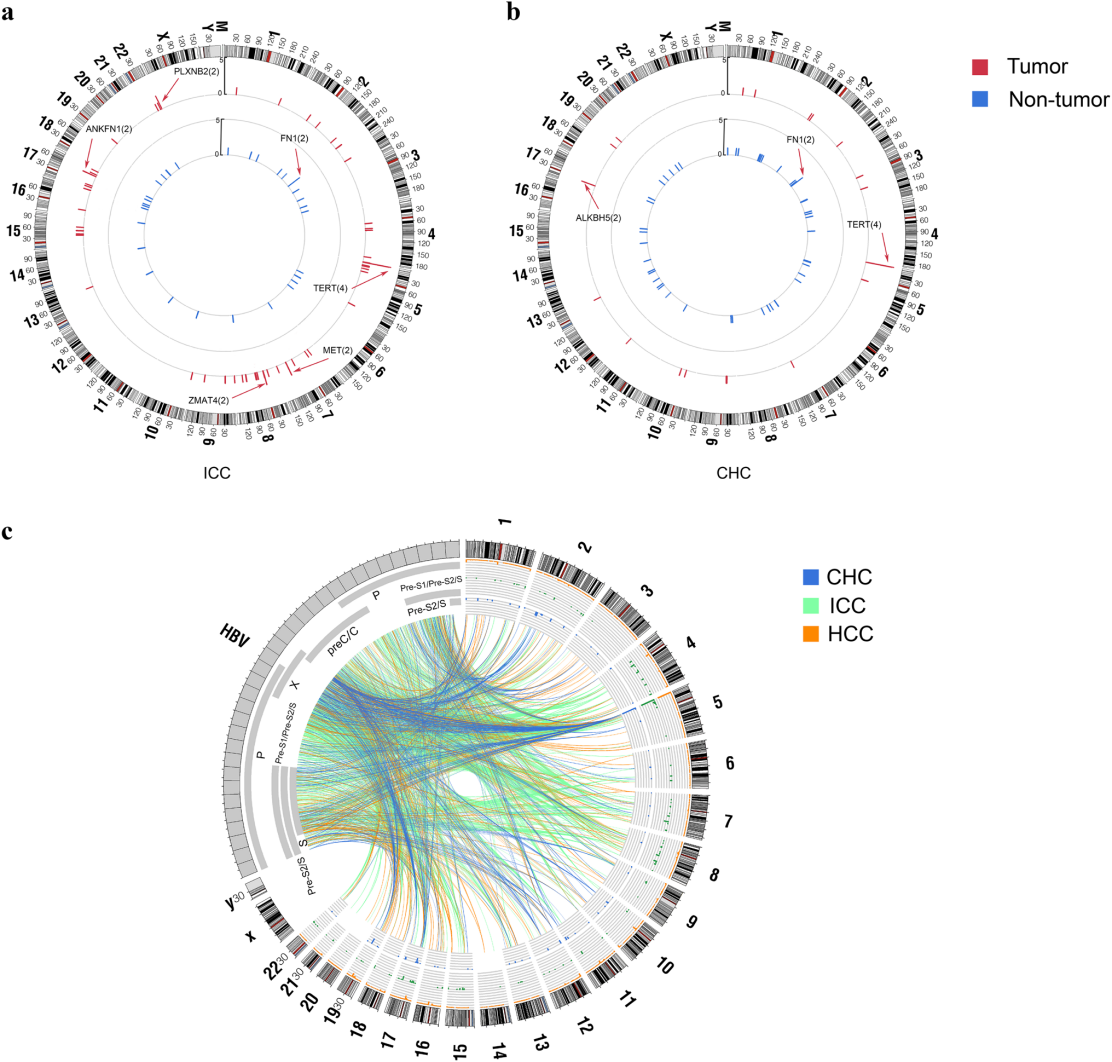
HBV integration breakpoints across human genome. **a** Sample frequency of integration breakpoints in ICC patients; **b** sample frequency of integration breakpoints in CHC patients; each bar represents the sample frequency of HBV integration breakpoints at a particular locus in the human genome (hg38). Tumor (red) and non-tumor (blue) samples with HBV integrations are shown on the inner and outer circles, respectively. Histogram axis units represent number of samples. Some loci with a high frequency of integration are marked. **c** The distribution of breakpoints on human genome and their corresponding source location on HBV genome

To investigate the function of HBV integrations in our samples, we analyzed the distribution of HBV integration sites across the genome. As a result, we found that HBV integration might prefer to affect the CDS (coding sequence) region of the human genome in CHC tumor samples. And the frequency of HBV integrations in tumor tissue of CHC patients was also significantly higher in intergenic region than in non-tumor tissue (Fig. S1), suggesting that HBV is likely to affect the gene function through integrating into coding region (CDS) and intergenic regulating region. To study the function of the HBV integrations in tumor tissues and adjacent non-tumor tissues, we made an annotation for the HBV integration sites. There are 44 genes with HBV integrations in tumor tissues and 30 genes in non-tumor tissues. Further analysis indicated that HBV tended to integrate into a few hot spots in tumor samples in both ICC and CHC, while in non-tumor tissues, the HBV integrations tend to be scattered across the genome (Fig. 2). This result indicated that HBV integration in some target genes might play etiological role in tumorigenesis. Notably, the data of both ICC and CHC indicated a recurrently integrated genes FN1 in non-tumor tissue, which was consistent with previous research on HCC [24, 25], suggesting that the adjacent non-tumor tissues of all three PLC types had the analogous profile of HBV integration. In ICC patients, the genes with HBV integrations in more than 1 tumor samples were *TERT* (4), *ZMAT4* (2), *MET* (2), *ANKFN1* (2), and *PLXNB2* (2); while in non-tumor tissues, only *FN1* contained HBV integrations in 2 samples, which is consistent with the results of our previous study of HCC [24]. For CHC patients, *TERT* and *ALKBH5* are found to be integrated by HBV fragments in 4 tumors and 2 tumors respectively; interestingly, *FN1* is also the only gene with HBV integration event occurred in more than 2 non-tumor samples, which is consistent with the results of ICC samples (Fig. 2a, b). When calculated by number of integration events, we found that *TERT* still had the high number of integration sites in tumors of both ICC (27) and CHC (22). While the genes with second highest number of HBV integration were *HAUS5* (8) and *ANKFN1* (14), respectively, in ICC and CHC, we observed that the landscape of HBV integration in the three subtypes of liver cancer showed significant differences (Fig. S2). Only a small number of insertion sites were shared by ICC, CHC, and HCC, underscoring that integration patterns are distinct in the three different types of primary liver cancer (Fig. S3) [24]. Besides, we also found that many breakpoints in human genome could be integrated by more than one HBV fragments; and in the same way, each HBV breakpoints may integrate into more than one location in human genome. Furthermore, HBV breakpoints occurred frequently at the end of X protein and the head of preC/C protein in all three types of tumor tissues. Besides, breakpoints in CHC also distributed in Pre-S1/S and Pre-S2/S region (Fig. 2c).

### Breakpoints on HBV genome

**Fig. 3:**
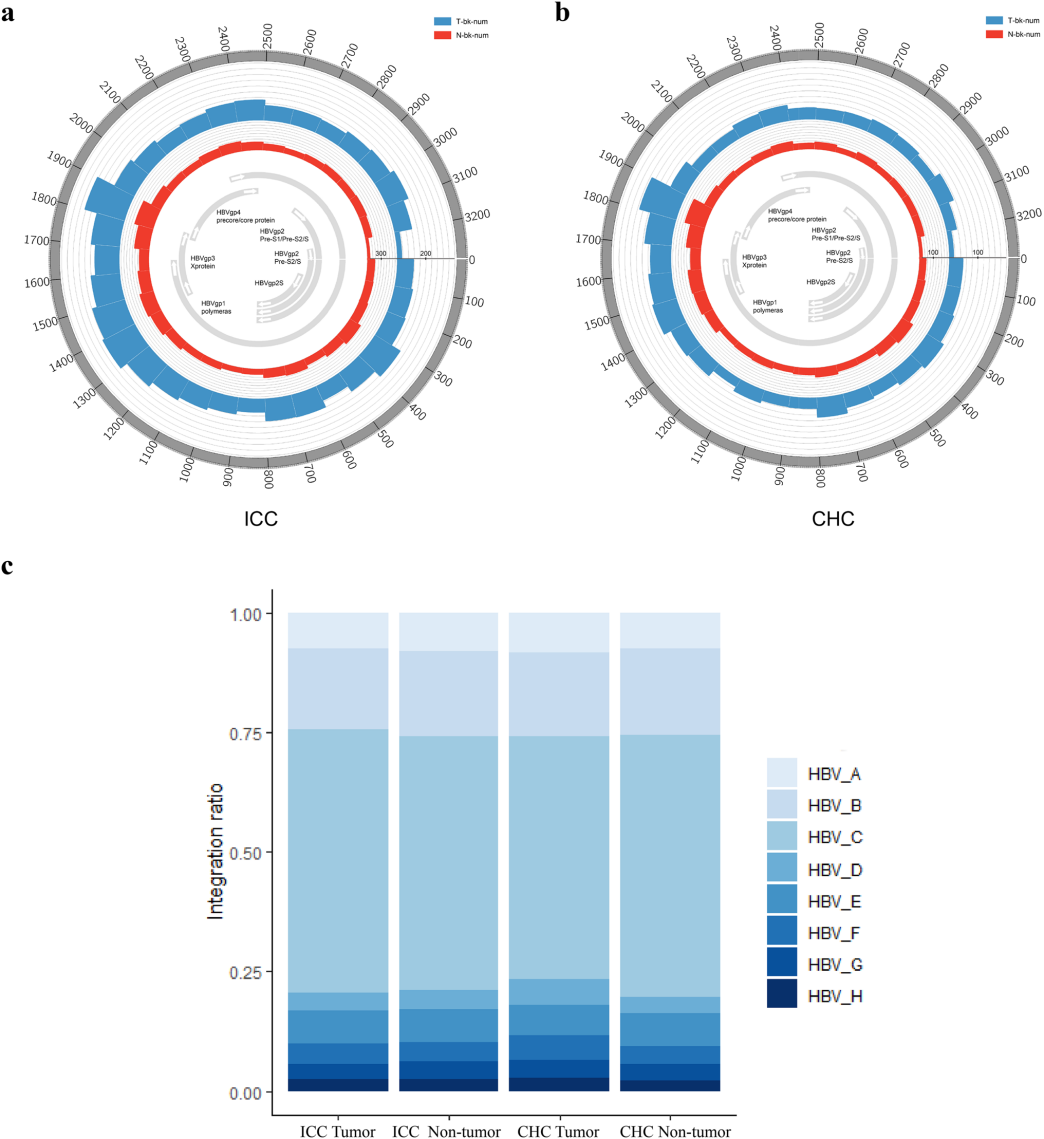
Integration breakpoints of HBV genome. **a** The breakpoints distribution in ICC patients. Histogram axis units represent number of breakpoints in 100-bp intervals. HBV genes and the orientation are shown in innermost circles. **b** The breakpoints distribution in CHC. **c** The HBV genotypes of integration breakpoints in tumor and non-tumor of ICC and CHC patients

To further investigate the interaction between host and virus, we also analyzed the distribution of the breakpoints on HBV genome. We found an enrichment of HBV breakpoints in the region of 1400–1900 bps containing the whole HBx gene and 5′end of the Precore/Core genes in both tumor and non-tumor samples of ICC and CHC tissues (Fig. 3a, b), which was similar to that of HCC tissue we reported before [24], suggesting that HBV virus might produce HBx protein through fully integrated HBx gene and maintain regulation promoter sequence of precore/core gene to facilitate its survival and replication in human cell. Previous studies also showed that the expression of HBx was a factor overcoming silencing of cccDNA or HBsAg expression as a possible modulator of the adaptive immune system. Furthermore, core and HBx proteins were potential candidates for direct involvement in the integration process due to their DNA-binding activities [32, 33]. In China, HBV genotypes B and C account for the majority of HBV carrier patients, with genotype B predominating in the central region and genotype C predominating southern and northern region [34, 35]. The main genotypes are genotypes B and C in ICC and CHC patients of our study, and genotype C accounted for more than 50% (Fig. 3c).

### Integration sites affect the genome instability

**Fig. 4:**
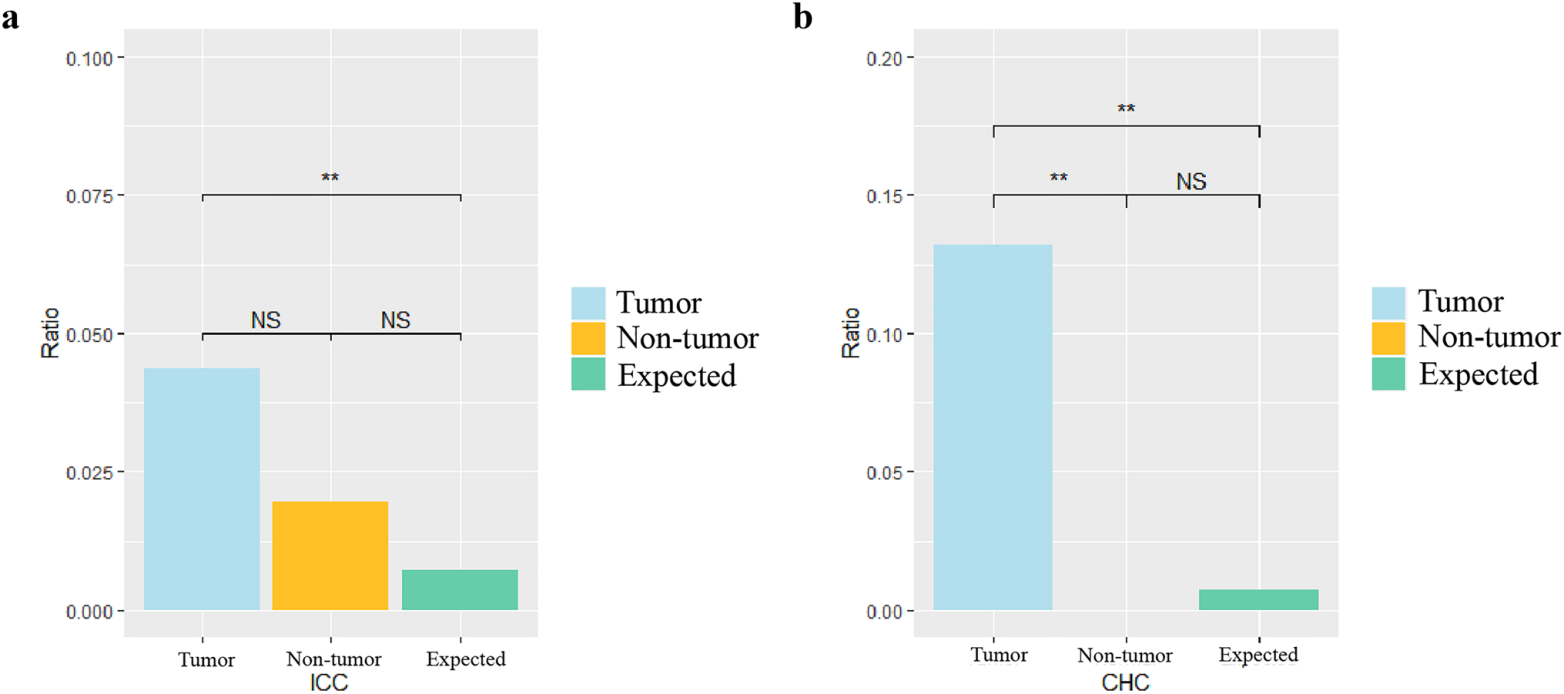
Comparison of the breakpoints in the CpG island region of 47 ICC and 20 CHC patients. *P* values were calculated by Chi-square test. The expected ratio is calculated based on random distribution

HBV affects the stability of the genome by integrating into the human genome, thereby causing cell carcinogenesis. CpG islands, which are closely related to genome stability, are mainly located in the promoter and exon regions near gene transcription regulatory regions [36]. Genomic and epigenetic regulations of CpG sites across the chromosomes play an important role in liver cancerization [37, 38]. In this study, we found that for both ICC and CHC patients, the HBV integrations in the tumor samples are significantly enriched in CpG island region compared to random distribution, indicating that HBV integration is not a random event, but could provide certain selection advantages for cancer cell. Furthermore, the occurrence of HBV integrations in CpG regions is significantly more frequent in tumor than in non-tumor tissues in CHC (Fig. 4), suggesting that HBV preferentially integrates into the specific regions which may affect the gene expression and regulation in cells and induce cell cancerization.

**Fig. 5:**
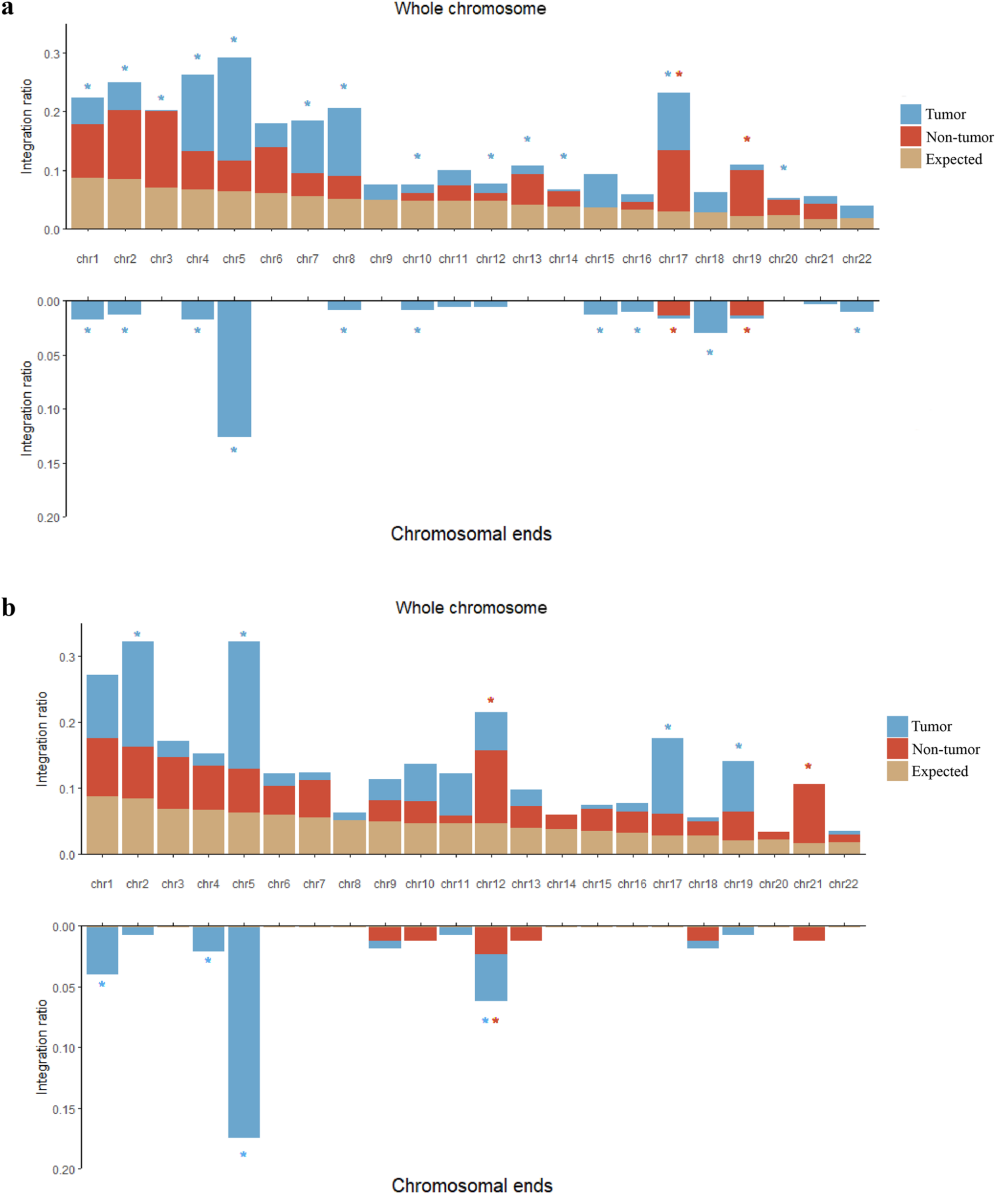
Whole chromosome and chromosomal ends enrichment or non-enrichment of HBV integration in ICC (**a**) and CHC (**b**). Each bar of whole chromosome represents the expected (assuming uniform, random distribution, expected in yellow) and the observed (actual numbers in tumor tissue: tumor in blue; non-tumor tissue: non-tumor in red) ratio of HBV integration breakpoints at a particular chromosome in human genome. Ratios are numbered. Each bar of chromosomal ends represents the expected (assuming uniform, random distribution, Expected in yellow) and the observed (actual numbers in tumor tissue: tumor in blue; non-tumor tissue: non-tumor in red) ratio of HBV integration breakpoints at the 2 M region of chromosomal ends in human genome. Ratios are numbered. Red star represents statistically significant difference between non-tumor liver samples and random distribution. Blue star represents statistically significant difference between tumor samples and random distribution. (*P* values < 0.05) *P* values were calculated by Chi-square test

Since HBV DNA could integrate into the human genome in the early stages of liver cancer, and it is reported that HBV may lead to chromosomal instability after integrated into HCC genome [24, 39, 40], we next analyzed the distribution of HBV breakpoints on chromosome level in our data. As a result, in ICC patients, the HBV integration events were significantly enriched in many chromosomes including chr 4, 5, 7, 8, 17 and non-enriched in chr 1, 2, 3, 10, 12, 13, 14, 20 in tumors; while in non-tumor tissues, only chromosomes 17 and 19 were enriched with HBV integrations (Fig. 5a). As for CHC patients, only chromosomes 2, 5, 17 and 19 are enriched for HBV integrations in tumors and chromosomes 12 and 21 are enriched for HBV integrations in non-tumor tissues (Fig. 5b). We observed that no common overrepresented chromosomes were found between ICC and CHC adjacent non-tumor tissues. In addition, this result is also distinct from the situation of HCC in our previous study where chromosomes 5, 16, 17 and 19 were enriched with HBV integrations [24]. Interestingly, as overrepresented chromosomes in HCC, two (chromosomes 5 and 17) were also enriched with HBV integrations in both ICC and CHC tumors.

As telomeres at chromosome ends play a critical role in genome stability, we then checked the distribution of integration sites in chromosome ends. As a result, there was a significant increase in the frequency of HBV integration at the telomeres in chromosomes 1, 2, 4, 5, 8, 10, 15, 16, 18 and 22 in ICC tumors, while only chromosomes 17 and 19 showed enrichment of HBV integrations in adjacent non-tumor tissues (Fig. 5a). While, in CHC tumor, HBV preferred to integrate into the ends of chromosomes 1, 4, 5 and 12, and only the ends of chromosome 12 were enriched for HBV integrations in adjacent non-tumor tissues (Fig. 5b). Most of the overrepresented chromosomes in CHC tumors were also overrepresented in ICC tumor (chr1, 4, 5), while no common overrepresented chromosomes existed for adjacent non-tumor tissue of ICC and CHC.

In summary, a relatively random distribution of integration sites is observed in non-tumor samples compared to tumor samples. The results also suggested that affected chromosomes/telomeres in tumors of CHC were a subset in that of ICC, which might be explained by that fact that CHC has some common pathological features of ICC and HCC. And the different profiles of HBV integrations in the non-tumor tissues of ICC and CHC suggested that the HBV integration events were randomly occurred in initiation stage as non-tumor liver tissue may be early status of tumor. These results underscoring that the preferential integrations might be closely related to maintain the stability of the genome.

### Analysis of RNA-seq and WGS data

**Fig. 6:**
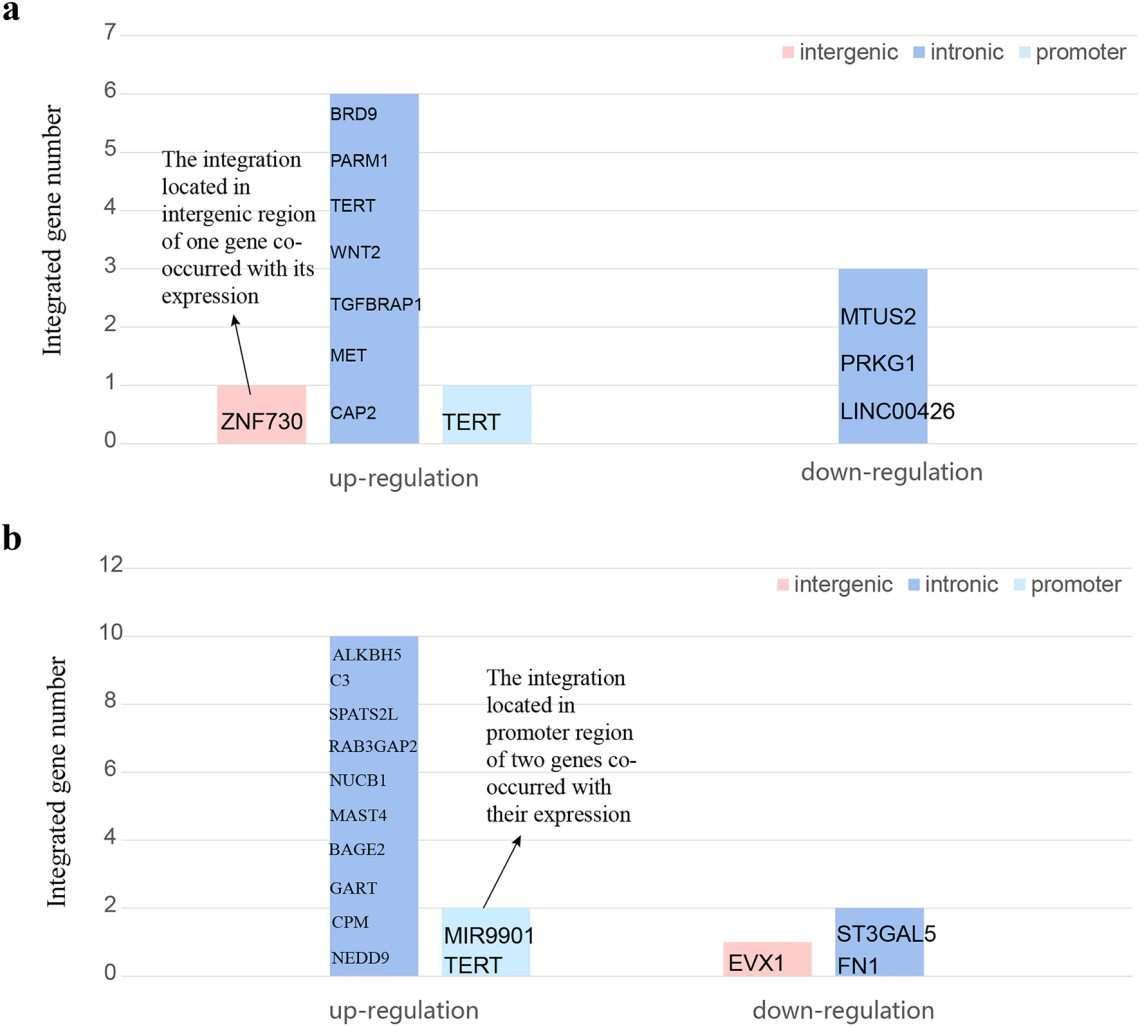
Change of expression level of integrated genes in **a** ICC and **b** CHC. Upregulated: tumor > non-tumor; down-regulated: tumor < non-tumor

To explore the effect of HBV integration on gene expression, we selected tumors and adjacent non-tumor liver tissues from three ICC and four CHC patients for RNA-seq and WGS. For RNA-seq data, we measured the level of gene expression and calculated the difference of the expression levels for each gene between tumor and non-tumor tissue (Table 1). As a result, in the samples of ICC patients, we found 11 integrated genes with differentially expressed between tumor and non-tumor tissue. Out of the 11 genes, 8 were up-regulated and 3 were down-regulated in tumors. Out of the 8 up-regulated genes, 2 were integrated in non-tumor samples, and 6 were integrated in tumor samples. 7 out of the 8 genes contained breakpoints in intron, one is in intergenic region and another one is in promoter region. On the other hand, there are 3 genes with breakpoints in non-tumor samples showing down-regulated expression level in tumor samples. All the 3 integrations occurred in intron (Fig. 6a). It is notable that *TERT* with integrations in both intergenic and promoter regions were up-regulated.

**Table 1:** Overview of the number of differentially expressed genes with HBV integrations

	ICC		CHC	
	Upregulated in tumor	Downregulated in tumor	Upregulated in tumor	Downregulated in tumor
Integrated in tumor	6	0	4	0
Integrated in non-tumor	2	3	8	3

In the samples of CHC patients, we found 15 differentially expressed genes with HBV integration between tumor and non-tumor tissues. Out of the 15 genes, 12 were up-regulated and 3 were down-regulated in tumor. Of the 12 genes, 8 contained HBV breakpoints in non-tumor tissues and 4 were in tumor tissues. When further digging deeper into the genome structure, we found that 10 out of the 12 breakpoints were in intron and 2 were in promoter. The 4 integration sites in 3 genes co-occurring with down-regulation of gene expressions were all located in non-tumor samples. Out of the 3 integrated genes, 2 contained HBV breakpoints in intron and 1 contained breakpoint in intergenic region (Fig. 6b).

In summary, most of the HBV integration were in intron. And in ICC, the genes with HBV integrations in tumor tend to be up-regulated and the gene down-regulated in non-tumor tissue has the correlation with HBV integration in non-tumor tissue, which means that no matter HBV integration occurs in tumor or non-tumor tissue, the integrations have the tendency to co-occur with the up-regulation of the corresponding tissue. And in both ICC and CHC, all genes with HBV integrations in tumor showed up-regulations. It is worth noting that only *TERT* contained HBV integration breakpoint in both ICC and CHC samples, and in both intron and promoter region.

Then, we focused on the up-regulated genes with HBV integration in tumor tissue. In ICC samples, the expression value of HBV-integrated genes, including *MET*, *WNT2*, *BRD9*, and *TERT*, in tumor seems to be higher than the paired non-tumor tissues. Consistently, the expression values of these genes in integrated tumor tissues are also likely higher than that of non-integrated tumor tissues (Figs. S4–S7). The results suggest that the expression of some viral integrated genes might be up-regulated due to the HBV integrations.

**Fig. 7:**
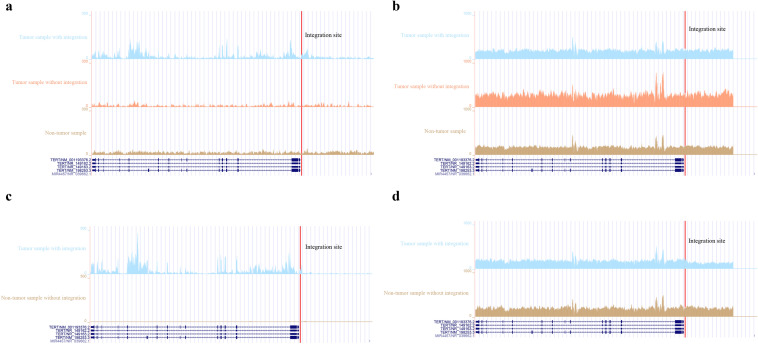
The effect of HBV integration, the genome variation, and transcription near TERT. **a** Differences in *TERT* gene expression between tumor tissues with HBV integration in CHC (tumor, upper panel) and adjacent non-tumor tissues without HBV integration (lower panel). **b** The difference in DNA copy number between tumors with HBV integration, tumors without HBV integration, and adjacent non-tumor tissues in CHC; **c** differences in *TERT* gene expression between tumor tissues with HBV integration in ICC (tumor, upper panel) and adjacent non-tumor tissues without HBV integration (lower panel). **d** The difference in DNA copy number between tumors with HBV integration and adjacent non-tumor tissues in ICC

To further explore the relationship between HBV integrations, gene expressions and genomic variations, we also analyzed the WGS data for the same batch of samples of RNA-seq. We found that the HBV integration in the oncogenic-related gene *TERT* in ICC could increase its expression level (Fig. 7c) and reduce downstream DNA copy numbers (Fig. 7d). This suggests that HBV fragments could integrate near the oncogene, causing large-scale genome variations on nearby genomic sequences, and, at the same time, changing the expression level of the oncogene genes. In CHC tissues, we also found that HBV integration could reduce DNA copy numbers and up-regulate the gene expression levels of *TERT* (Fig. 7a, b).

## Discussion

PLC can be categorized into three major histological subtypes: HCC, ICC, and CHC [20]. These three subtypes of PLCs are distinct with respect to epidemiology, clinicopathological features, genetic alterations, and clinical managements. And the association of HBV infections and the development of ICC or CHC still remain controversial. Our study presents an integrative analysis of HBV genomes in both non-tumor and tumor liver tissues of a large cohort of patients with ICC and CHC.

During the last few years, our knowledge of HBV integration has greatly improved owing to the development of new detection methods and research models. However, many key aspects of viral integration remain to be defined, such as the role of HBV integration in PLC initiation and development [41–43]. The origin of HCC, ICC, and CHC remains poorly understood, and all three subtypes of PLC have a high rate of hepatitis B infection, especially in China. Tumorigenesis is a multicausal and multifactorial process, in which many integration defects and gene mutations are involved. Of note, HBV integration occurs in the early stage of virus infection, and it might imply possible common virus-related mechanisms underlying the development of the primary hepatic neoplasias. Moreover, as supported by the relevant literatures, some target genes of HBV integration may participate in hepatocarcinogenesis [4, 24, 44–47]. It is noteworthy that although ICC and CHC are distinct molecular subtypes and histologically different, they both showed stem-like features and very poor prognosis. How the high stemness is maintained in both ICC and CHC remains an interesting, yet rarely explored question [48]. In our study, distinct patterns of HBV integration between the three PLC types and between tumor and non-tumor tissues suggest that HBV integrations during malignant transformation of hepatocytes might partially explain their pathological differences. Consistent with the observations in HCC, our results also revealed that HBV DNA has its preferential targets in both ICC and CHC. We detected multiple genomic HBV integrations in two distinct types of liver cancer, with recurrent events at *TERT, ZMAT4, MET, ANKFN1, PLXNB2* in ICC, and *TERT, ALKBH5* in CHC. These findings differ from those reported in HCC, although the *TERT* was a preferred site of viral integration in all three diseases. One previous study mentioned that replicating HBV DNA was also detected in non-tumor tissues and associated with a higher number of non-clonal integrations. As in tumor, clonal selection of HBV integrations was closely related to carcinogenesis [23]. These results suggested that although most HBV integrations are passenger events and do not have functional consequences, some might play a part in the PLC transformation process.

Notably, in the genomes of ICC and CHC from our data, *TERT* is repeatedly integrated by the HBV, which is highly consistent with our previous research in HCC [24]. This suggested that HBV DNA integration into the TERT might be a common factor in the initiation of various PLC types. To our knowledge, the role of HBV integrations into *TERT* in the initiation and development of both ICC and CHC has never been systematically documented before. Whether the common HBV integration pattern in *TERT* is associated with similar stem-like features and poor prognosis in the two PLC types remains to be explored. Although it remains uncertain whether human ICC and CHC also arise from hepatocytes, many previous studies provided answers to the question of why patients with viral hepatitis often develop ICC and CHC [14, 49, 50]. Our data suggested that the activation of *TERT* by HBV insertion may play an important etiological role of tumorigenesis of HCC, ICC, and CHC. We speculate that the recurrent HBV integrations together with other oncogenic events including mutations on some key genes could be lineage conversion during malignant transformation of hepatocytes. Our findings demonstrate that multiple HBV-related PLCs may share a common HBV integration-based mutagenic origin in hepatocytes malignant transformation. However, how ICC and CHC initiated and developed in the regulation of HBV integration and other genome variations remains an interesting question and warrants future studies.

To the best of our knowledge, our study is the first study to investigate and compare the comprehensive patterns of HBV integrations among three major histological subtypes of PLC. Moreover, there have been few genomic studies on HBV-related ICC integration sites before, and even if there are, only some HBV integration targets are briefly mentioned. In addition to frequent integrations at *TERT* and *FN1* which were known targets of HBV insertion, we also discovered many new recurrent HBV integrations in ICC and CHC patients. Our data greatly expand the list of affected genes by HBV integration, providing an important amendment to the understanding of HBV impact on hepatocarcinogenesis of both HBV-related ICC and CHC.

Our in-depth analysis has demonstrated numerous mutational events in ICC and CHC resulting from HBV insertions in the host genome. We provided a detailed genomic landscape of HBV insertion events and a comprehensive comparison of HBV integration in the two most common subtypes of hepatic malignancy after HCC. We have reported the preferences of HBV integrations in human genome, recurrently integrated genes, and their interactions on copy-number variation (CNV) and gene expression. Together with the integrations of HBV genome, the characteristics including high stemness of liver tissue may endow a greater opportunity to induce crucial hepatocytes’ malignant transformation of host genes and eventually leading to PLC development in patients with chronic HBV infection. In conclusion, our data greatly expand the list of affected genes by HBV integration, providing an important amendment to the understanding of HBV impact on hepatocarcinogenesis of both HBV-related ICC and CHC.

## Supplementary Information

Below is the link to the electronic supplementary material.

Supplementary file1 (DOCX 44656 KB)

Supplementary file2 (PDF 68 KB)

Table S2. HBV integration sites in tumor of ICC (XLSX 43 KB)

Table S3. HBV integration sites in non-tumor tissue of ICC (XLSX 16 KB)

Table S4. HBV integration sites in tumor of CHC (XLSX 21 KB)

Table S5. HBV integration sites in non-tumor tissue of CHC (XLSX 16 KB)

Table S6 HBV DNA integration breakpoint number and supporting reads number per breakpoint in ICC (XLSX 12 KB)

Table S7 HBV DNA integration breakpoint number and supporting reads number per breakpoint in CHC (XLSX 11 KB)

## Data Availability

All data generated or analyzed during this study are included either in this article or in the supplementary information files.

## Abbreviations

ICC: Intrahepatic cholangiocarcinoma
HCC: Hepatocellular carcinoma
CHC: Combined hepatocellular-cholangiocarcinoma
HBV: Hepatitis B virus
TERT: Telomerase reverse transcriptase
ZMAT4: Zinc finger matrin-type 4
ANKFN1: Ankyrin repeat and fibronectin type III domain containing 1
PLXNB2: Plexin-B2
ALKBH5: AlkB homolog 5
HIVID: High-throughput viral integration detection
EHBH: Eastern Hepatobiliary Surgery Hospital
WGS: Whole-genome sequencing
RNA-seq: RNA sequencing
CNV: Copy-number variation
