# Supplementary material for: Analysis of viral integration reveals new insights of oncogenic mechanism in HBV-infected intrahepatic cholangiocarcinoma and combined hepatocellular-cholangiocarcinoma
Source: Hepatol Int. 2022 Sep 20;16(6):1339–52. doi: 10.1007/s12072-022-10419-3 (PMC9701178; doi:10.1007/s12072-022-10419-3)
Supplement: Supplementary file 1 — Supplementary file1 (DOCX 44656 KB) [file 12072_2022_10419_MOESM1_ESM.docx]

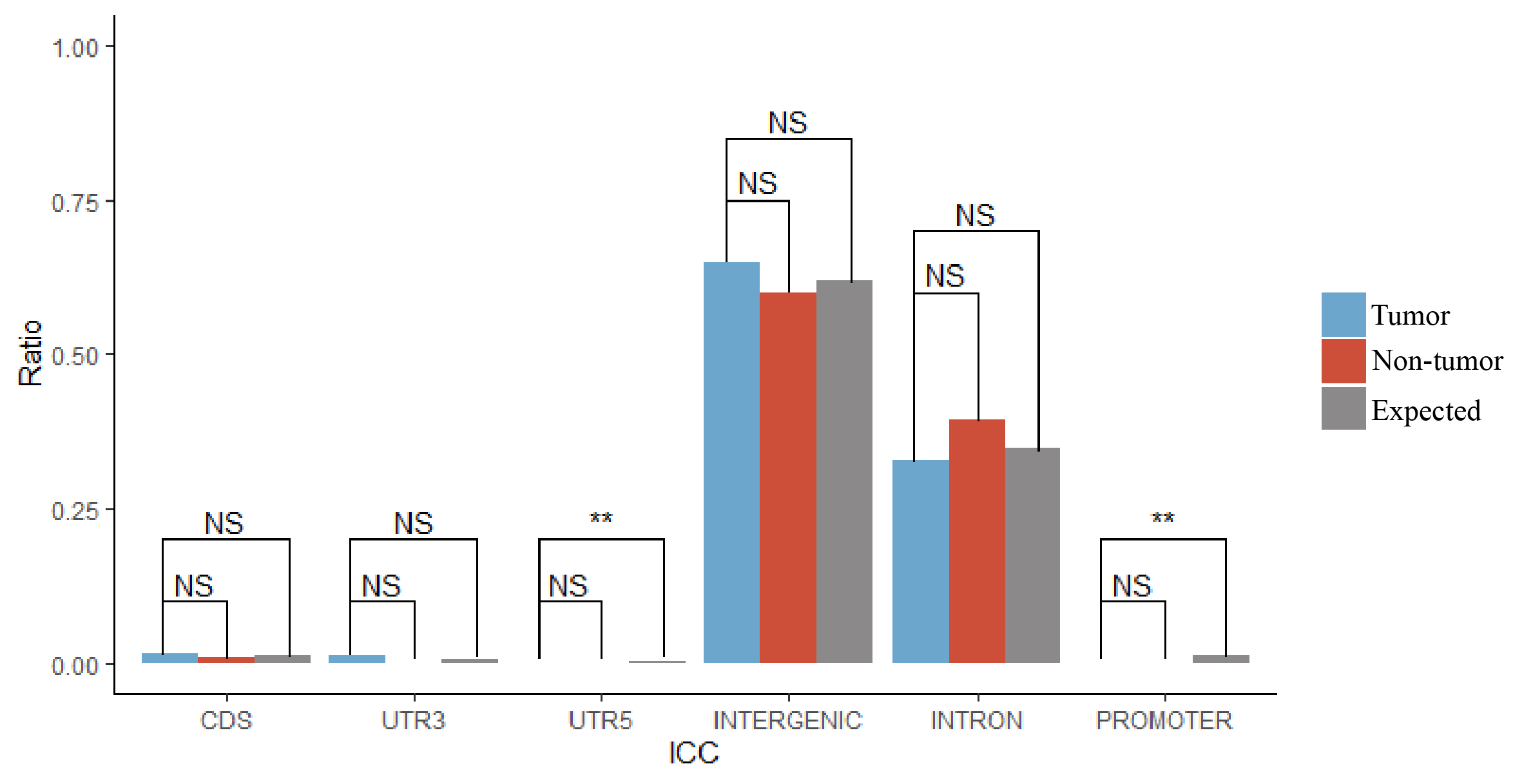


B

A


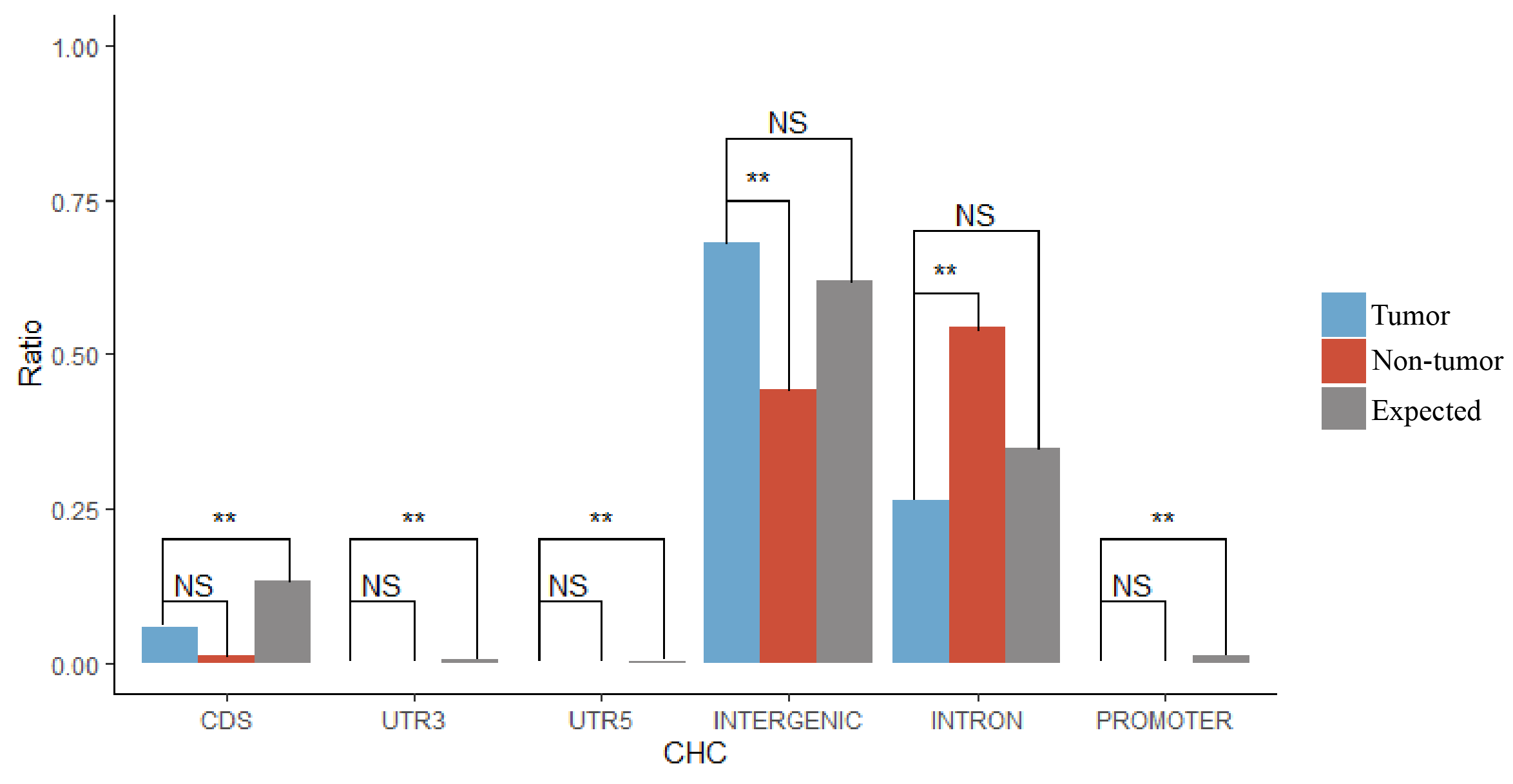


**Fig S1. The preference of HBV integration across the host genome elements. (A) ICC; (B) CHC.** The number of integrated breakpoints in each genome element was calculated. A uniformly random distribution of breakpoints across the entire human genome was used to calculate the expected ratio of breakpoint number. Grey bar shows the expected ratio of HBV-integrated breakpoints. Blue bar shows the observed ratio of HBV-integrated breakpoints in tumor tissues. Red bar shows the observed ratio of HBV-integrated breakpoints in non-tumor tissues. P values were calculated by Chi-squared test, ** means P values <0.05.


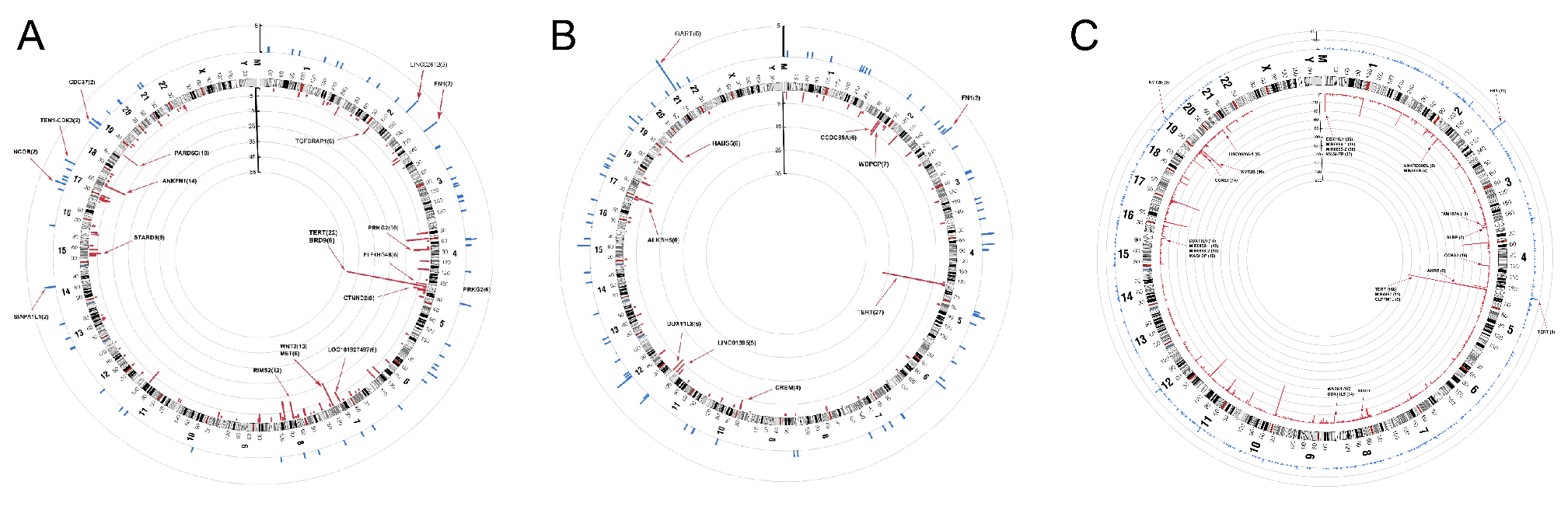
**Fig. S2. HBV integration hot spots calculated by the number of breakpoints in sliding windows in human genome (hg19; interval size: 1 Mbps).** Red bars of inner circle indicate integration breakpoints number of tumor samples. Blue bars of outer circle indicate integration breakpoints number of paired non-tumor samples. Some loci with a high frequency of integration in intragenic region of gene are marked. (A) ICC; (B) CHC; (C) HCC

**Figure S3. Overlap of integration related gene in tumor and non-tumor samples of ICC, CHC and HCC.** Left panel show the overlapped gene number of integrated gene. Horizontal histogram on the left represent the summarize breakpoints number of each sample group. The right histogram indicate the overlapped gene number of groups presented in beneath with dot and line. Venn plot in right panel also represents overlapped number of integrated gene. (A) Overlap of all integrated gene. (B) Overlap of recurrent integrated gene which was discovered in more than one sample


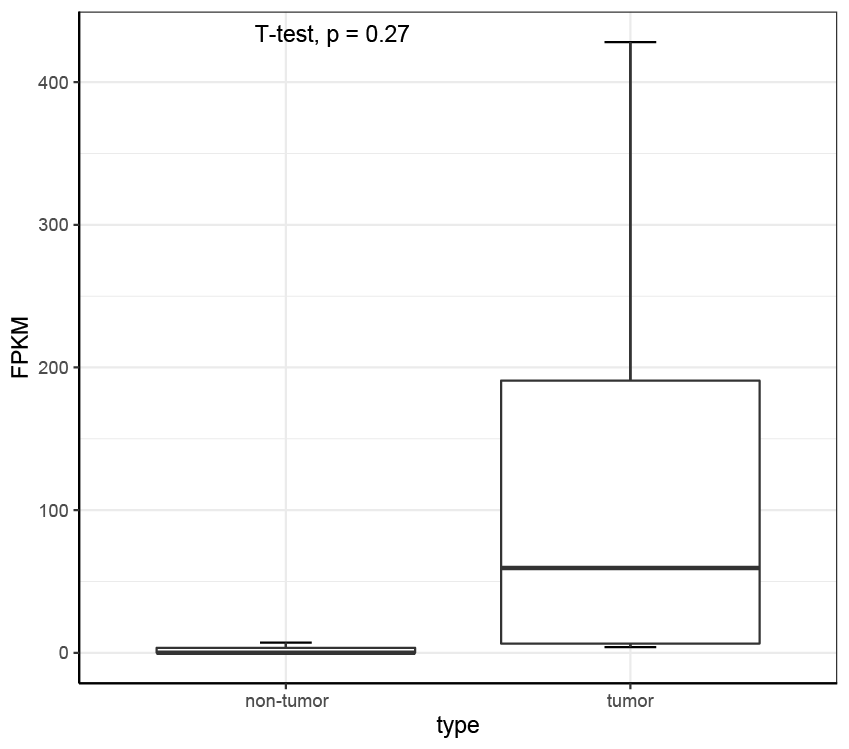

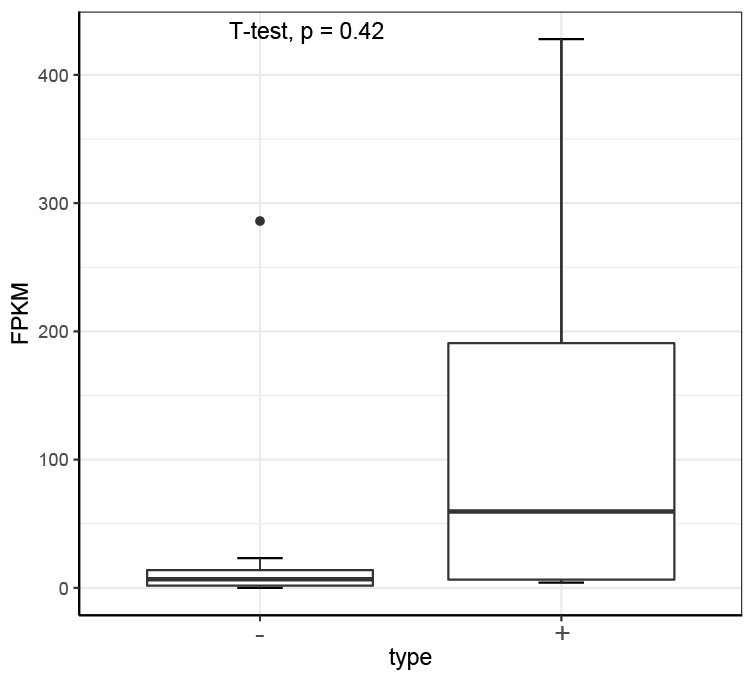


**Fig S4. FPKM (****Fragments per kilobase of transcript per million fragments mapped) of MET in ICC samples.** Non-tumor indicates FPKM in non-tumor tissue. Tumor indicates FPKM in tumor tissue. The (-) indicates non-integrated tumor tissues of this gene. The (+) indicates integrated tumor tissues of this gene. P values were calculated by Student's t-test.


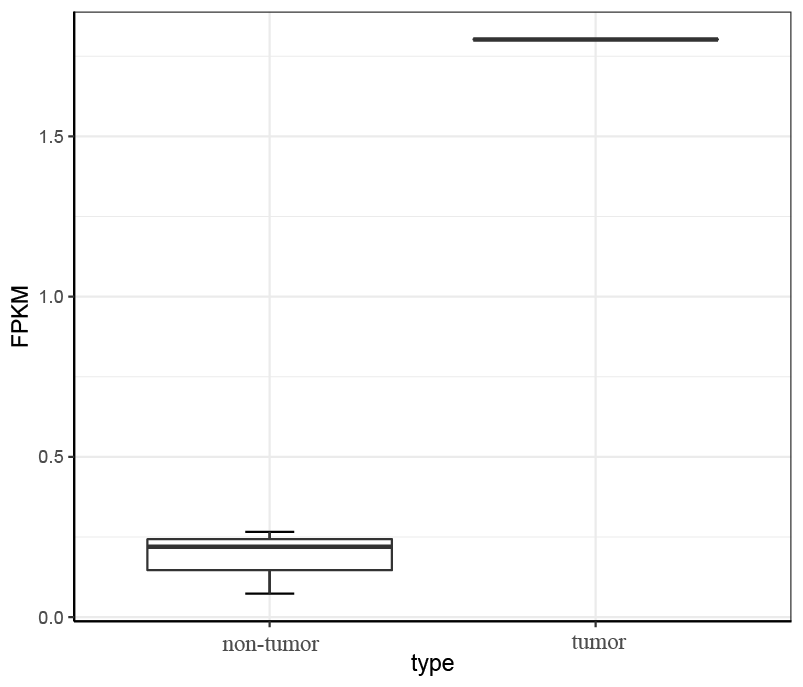

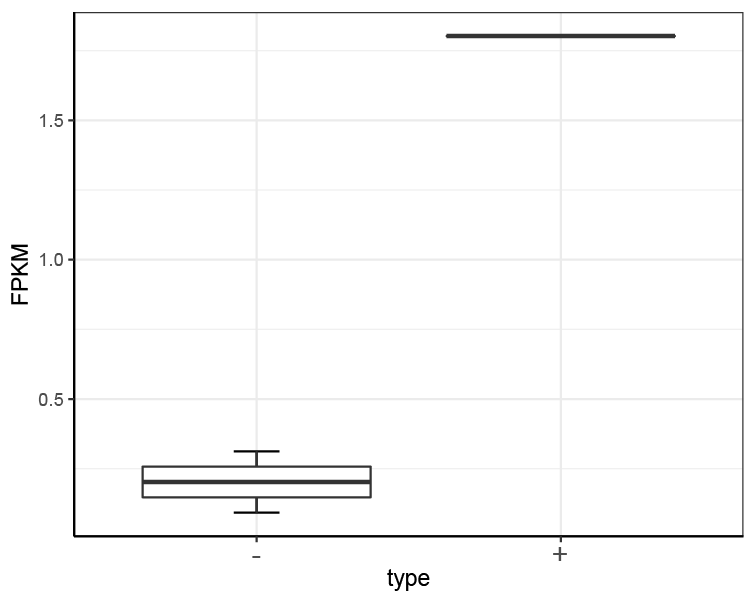


**Fig S5. FPKM of WNT2 in ICC samples.** Non-tumor indicates FPKM in non-tumor tissue. Tumor indicates FPKM in tumor tissue. The (-) indicates non-integrated tumor tissues of this gene. The (+) indicates integrated tumor tissues of this gene. P values were calculated by Student's t-test.


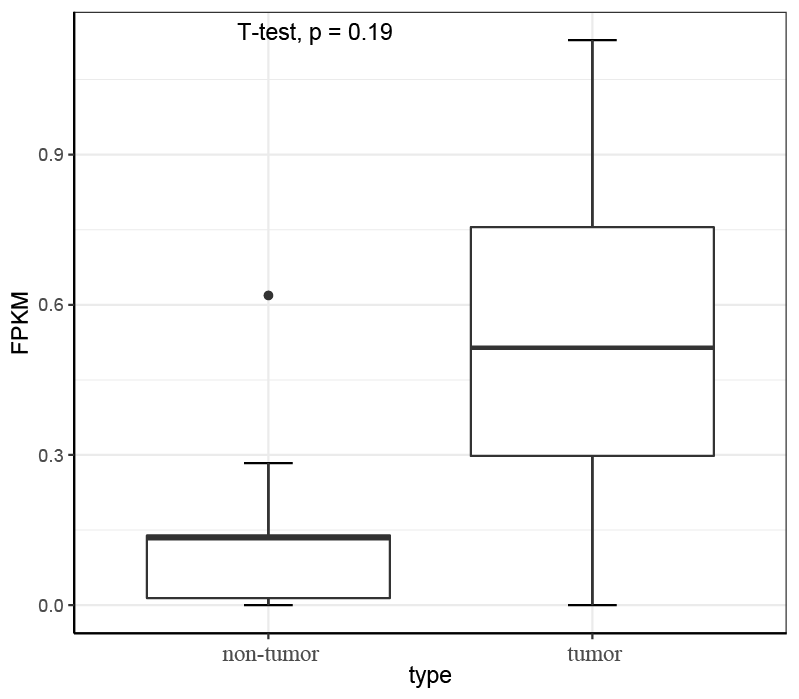

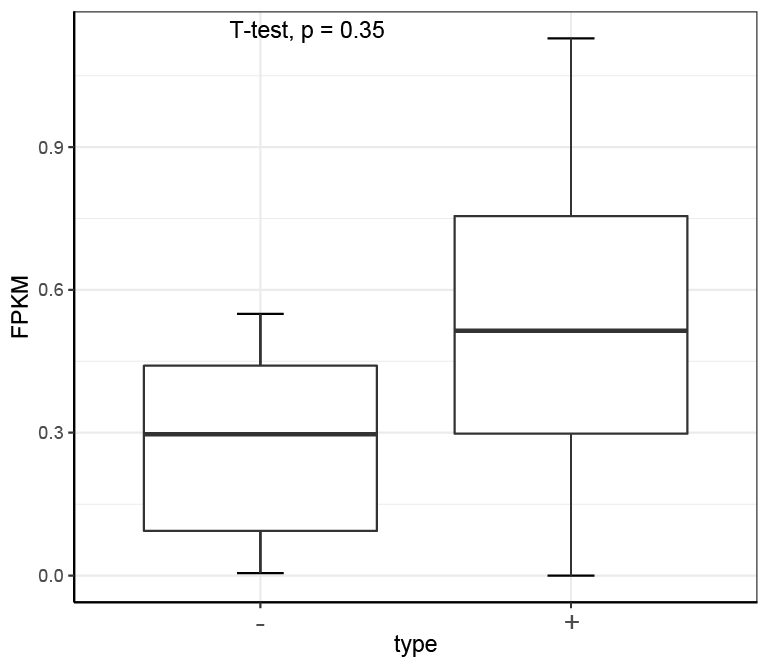


**Fig S6. FPKM of BRD9 in ICC samples.** Non-tumor indicates FPKM in non-tumor tissue. Tumor indicates FPKM in tumor tissue. The (-) indicates non-integrated tumor tissues of this gene. The (+) indicates integrated tumor tissues of this gene. P values were calculated by Student's t-test.


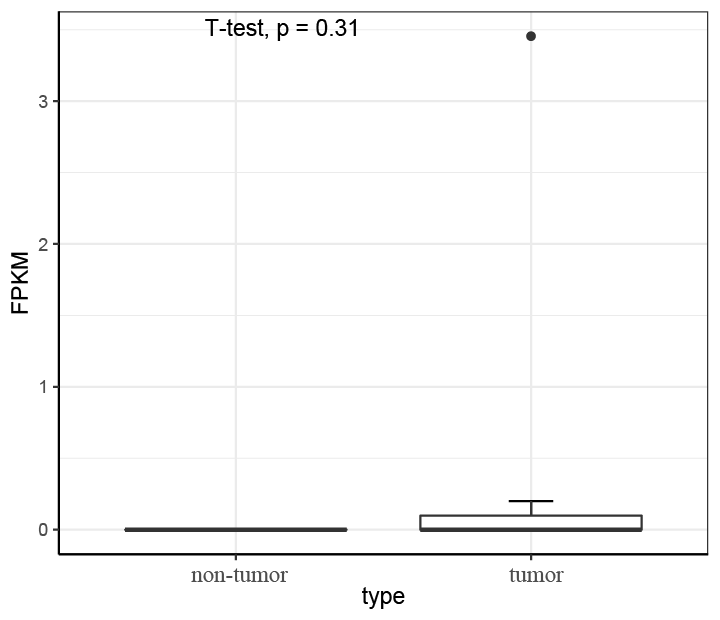

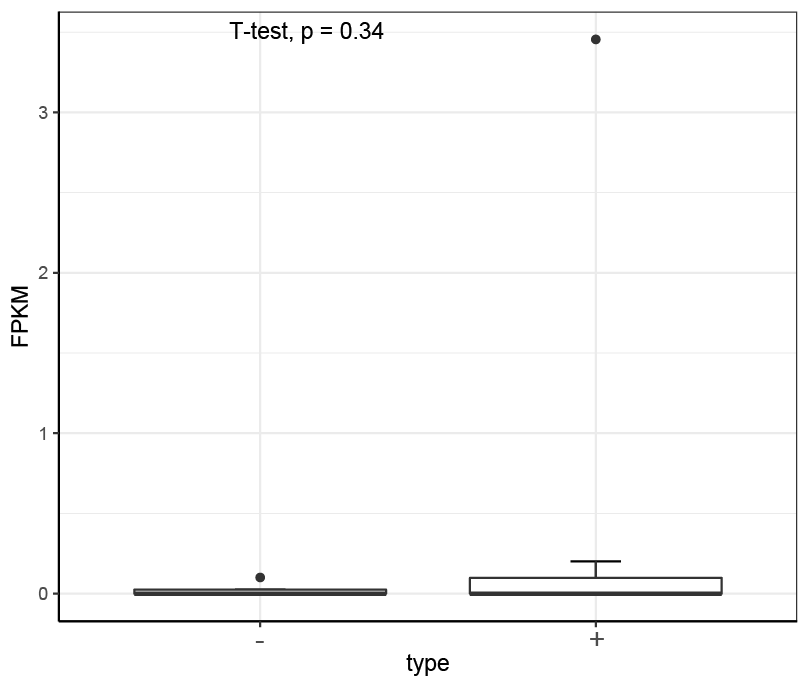


**Fig S7. FPKM of TERT in ICC samples.** Non-tumor indicates FPKM in non-tumor tissue. Tumor indicates FPKM in tumor tissue. The (-) indicates non-integrated tumor tissues of this gene. The (+) indicates integrated tumor tissues of this gene. P values were calculated by Student's t-test.
