# Supplementary material for: Analysis of viral integration reveals new insights of oncogenic mechanism in HBV-infected intrahepatic cholangiocarcinoma and combined hepatocellular-cholangiocarcinoma
Source: Hepatol Int. 2022 Sep 20;16(6):1339–52. doi: 10.1007/s12072-022-10419-3 (PMC9701178; doi:10.1007/s12072-022-10419-3)
Supplement: Supplementary file 2 — Supplementary file2 (PDF 68 KB) [file 12072_2022_10419_MOESM2_ESM.pdf]

Table S1. Demographic and clinicopathologic characteristics of ICC and CHC patients

Variables	Patients	
	ICC (N=41)	CHC (N=20)
Age(years)		
Mean(SD)	53.73(9.08)	51.10(11.13)
Sex, n (%)		
Male	25(60.98)	16(80.00)
Female	16(39.02)	4(20.00)
Cirrhosis, n (%)		
Non	17(41.46)	0(0.00)
Slight	16(39.02)	6(30.00)
Moderate	5(12.20)	12(60.00)
Severe	3(7.32)	2(10.00)
Tumor Capsular Integrity, n (%)		
Non-intact	36(87.80)	17(85.00)
Intact	5(12.20)	3(15.00)
ALB, g/L		
Mean(SD)	41.40(3.51)	39.17(3.41)
TBIL, μmol/L		
Mean(SD)	13.01(5.58)	12.76(4.44)
PT, seconds		
Mean(SD)	12.07(1.07)	12.90(0.93)
ALT, IU/L		
Mean(SD)	52.53(46.49)	48.95(48.43)
AFP ,ng/mL		
Mean(SD)	98.74(279.54)	196.20(320.22)
CA19-9, U/mL		
Mean(SD)	246.75(402.23)	46.74(94.67)
HBV DNA, copies/mL, n (%)		
Mean(SD)	404399.4(1010826)	95704.55(207618.2)
NA	5(12.20)	0(0.00)
<1000	22(53.66)	9(45.00)
1000-10000	2(4.88)	3(15.00)
10000-100000	6(14.63)	4(20.00)
>100000	6(14.63)	4(20.00)
HBsAg, n (%)		
Negative	5(12.20)	0(0.00)
Positive	36(87.80)	20(100.00)
HBeAg, n (%)		
Negative	30(73.17)	15(75.00)
Positive	11(26.83)	5(25.00)
